# SOX1 Antibody in a Patient With Serotonin Syndrome

**DOI:** 10.7759/cureus.48516

**Published:** 2023-11-08

**Authors:** Julia Hoffer, William Frem, Jessica Alkana, Charisse Chih, Antonio K Liu

**Affiliations:** 1 Internal Medicine, Adventist Health White Memorial, Los Angeles, USA; 2 Neurology, Adventist Health White Memorial, Los Angeles, USA; 3 Neurology, Loma Linda University School of Medicine, Loma Linda, USA

**Keywords:** immunomodulation, encephalitis, autoimmune, serotonin syndrome, sox1

## Abstract

SOX1 antibody is an autoimmune antibody, usually associated with Lambert-Eaton myasthenic syndrome, paraneoplastic conditions, and encephalitis. This antibody has also been found among psychiatric patients. However, the role of SOX1 antibody in serotonin syndrome has not yet been defined, as a literature search yielded no results. Therefore, the treatment as such has unknown clinical significance. In this case study, we report a patient with SOX1 antibodies and altered mental status out of proportion to serotonin syndrome whose symptoms improved with simultaneous treatment of both conditions.

## Introduction

Serotonin syndrome (SS) is a collection of mild to severe symptoms that arise from excessive serotonergic agonism of central nervous system receptors and peripheral serotonergic receptors [1]. Multiple receptors have been identified as potential players, such as 5-HT2A and 5-HT1A. These symptoms are characterized by autonomic dysfunction, neuromuscular excitation, and altered mental status. They can manifest as mild symptoms or in extreme cases can lead to death A large number of medications and street drugs, taken alone or in combination, can cause SS. Antidepressants that have been linked to SS include monoamine oxidase inhibitors (MAOI), selective serotonin reuptake inhibitors (SSRIs), tricyclic antidepressants, trazodone, and others. Central nervous system stimulants such as 3,4-methylenedioxymethamphetamine, amphetamine, cocaine, and methylphenidate are also common causative agents. Other agents that can cause SS include opioids, triptans, lithium (Li), linezolid, risperidone, olanzapine, ondansetron, ritonavir, and many others. Special attention must be paid to finding the offending agents [2]. One of the clinical tools in diagnosing SS is the Hunter serotonin toxicity criteria, which has a sensitivity of 84% and specificity of 97% [3].

SRY-box transcription factor 1 is a protein-coding gene that encodes a transcription factor in the DNA-binding domain and functions primarily in neurogenesis and cell fate determination [4]. SOX1 autoimmune antibodies have traditionally been associated with paraneoplastic neurological disorders such as Lambert-Eaton myasthenic syndrome (LEMS), cerebellar degeneration [5,6], and encephalitis [7]. Additionally, there is recent supporting evidence suggestive of its association with multiple sclerosis, motor neuron disease, Guillain-Barré polyneuropathy, and other neuroimmunological disorders without an underlying neoplasm [8]. In cases of autoimmune encephalitis, SOX1 has been identified as a target, and antibodies are discovered in both serum and cerebrospinal fluid [9]. Therefore, immune therapy such as intravenous immunoglobulin (IVIG), steroids, and rituximab may be utilized in these cases. Interestingly, there is initial data that antineuronal autoantibodies such as SOX1 are also discovered in a small percentage of patients with psychiatric conditions such as schizophreniform and affective psychosis [10-12]. However, there is no published correlation of SS with SOX1 antibody. In this case study, our patient had SS that satisfied the Hunter serotonin toxicity criteria and SOX1 positivity suggesting a possible link between these conditions and providing insights into treatment management.

## Case presentation

A 42-year-old male with hypertension, dyslipidemia, schizophrenia, autism, and developmental delay presented to our emergency department (ED) with his mother due to diffuse muscle stiffness. He was recently admitted to the mental health unit for acute psychosis and was discharged three days prior. During the previous admission, his medication was changed from olanzapine to risperidone and then Haldol. Furthermore, according to medical records, trazodone and ondansetron were used on an as-needed basis. There was no recent SSRI, MAOI, or Li use. The patient’s mother claimed they did not receive the prescription for benztropine (commonly prescribed with Haldol); therefore, the patient was only taking Haldol since discharge. He became increasingly stiffer, had difficulty with gait and swallowing, and was unable to open his jaw. Haldol was stopped before admission. Before all these, at baseline, he was able to ambulate, perform very basic activities of daily living, and even occasionally work at a sheltered site.

During the most recent ED visit, the patient presented with diffuse hypertonicity and appeared contracted. He was also slightly tremulous and had an inducible clonus with a light finger tap at the knees and ankles. He was agitated and oriented to name and location. His cranial nerves were normal without any ocular clonus or abnormalities. There was diaphoresis and hyperreflexia. The patient was hypertensive and had spiking fevers up to 38.5°C. Lab work revealed an elevated creatinine kinase (CK) of 560 IU/L. There were no focal neurological findings, and he had no rigidity. Urine toxicology results were repeatedly negative. All other chemistry and hematology results were noncontributory. His condition fulfilled the Hunter serotonin toxicity criteria.

Treatment for SS was initiated with oral cyproheptadine 2 mg three times a day and he gradually improved. His hypertonicity and clonus were resolving allowing him to move his extremities, ambulate, eat, and speak. There was no more agitation and diaphoresis. However, after three to four days of admission, he became more lethargic, disoriented, and altered in mental status. MRI of the head was noncontributory. Electroencephalography showed generalized slowing without an epileptic event. Spinal fluid analysis showed a cell count of 3 white blood cells/mm^3^ (range: 0-8) and protein of 25 mg/dL (range: 15-45). All spinal fluid bacterial, viral, and fungal studies were negative. The serum autoimmune neurologic diseases reflexive panel, which tested for 20 antibodies, came back positive for SOX1 (no value was given, reference was negative). A 10-day course of intravenous solumedrol 1,000 mg daily and a five-day course of IVIG 35 g daily were given. The patient’s mental status began to improve. After a seven-week hospitalization, the patient was discharged home after returning to baseline. He was readmitted six months later for an unrelated medical issue and had no neurological complaint or finding. Repeat testing revealed absent SOX1.

## Discussion

We reported a patient with a psychiatric background who developed SS. He fulfilled the Hunter serotonin toxicity criteria (clonus, agitation, diaphoresis, tremor, hyperreflexia, hypertonicity, and maximum temperature of above 38°C) (Table 1) [3]. Differential diagnosis at that point of the hospitalization also included neuroleptic malignant syndrome. However, his CK peaked at 560 IU/L, and he never developed lead pipe rigidity. Even though the patient did not receive SSRI or MAOI, he was exposed to risperidone, olanzapine, ondansetron, and trazodone [1].

**Table 1:** Hunter serotonin toxicity criteria.

Exposure to the serotonergic agent and one of the following:
Spontaneous clonus
Inducible clonus and agitation or diaphoresis
Ocular clonus and agitation or diaphoresis
Tremor and hyperreflexia
Hypertonic and temperature >38°C and ocular clonus or inducible clonus

After removing the causative agents, the patient responded to cyproheptadine and supportive care. With SS symptoms improving with cyproheptadine, however, his mentation worsened. Without any other etiologies discovered, an autoimmune neurologic antibodies panel was sent and SOX1 came back positive. There is no known association between SS and SOX1-related disorder. The discovery of SOX1 led us to initiate immunological modulation therapy and the patient recovered within weeks. At the six-month follow-up, the patient had not reported a recurrence of either condition.

We report a single case of SS with positive serum SOX1 at our center. While far beyond the scope of this report to discuss the cause and effect, we found no available prior published cases that were similar. There are several possible scenarios for our finding of SOX1 with SS. Besides the fact that this can be unrelated/incidental, SOX1 is known to have false positives during testing. Second, as we are trying to suggest in this paper, more studies will be needed to determine if SOX1 plays a role in SS or other psychiatric diagnoses. Third, SOX1 may be part of an overlapping syndrome or spectrum of disorders. Finally, SOX1 autoimmune encephalitis may have been triggered by other disease entities such as SS.

When testing for SOX1, one study proposed that multiple tests, i.e., immunofluorescence with either or both line blot or cell-based assays, should be used to confirm their presence as it is well recognized that there are limitations in testing [13] as well as false-positive results [11]. A variety of factors can lead to false positives, one of which may be the peroxidase used [14]. However, SOX1 may also be an incidental finding such as the antinuclear antibody that is present in around 2.5% of those who do not exhibit symptoms or have autoimmune diseases [15]. SOX1 antibodies have been linked to neoplastic, paraneoplastic, and non-neoplastic conditions with varying degrees of neurological involvement. Onconeural antibodies, specifically anti-Yo and anti-RI, have been found in those with psychiatric disorders [16,17]. These antibodies can also cause neurological damage by showing affinity to various parts of the central nervous system, leading to dysfunction secondary to inflammation. A study that examined 992 patients established that SOX1 was found in 2% of schizophreniform as well as 2% of patients with affective psychosis [10]. While rare and inconclusive, their presence is not uncommon.

Recent research has made inroads in establishing autoimmune contributing factors of psychiatric conditions [11,12]. A Danish study found the chance of schizophrenia in people with autoimmune diseases can be enhanced by 45% [18]. The role of antibodies in acute psychiatric presentations has been recorded in prior studies, and Pollock et al. propose it may play a role in diagnosis or treatment; hence, it is reasonable to consider a possible role of SOX1 in this domain. In the last few years, diagnostic and management criteria have been created for psychosis due to autoimmunity [19]. Additionally, treatment with IVIG may also extend to psychiatric presentations as IVIG was successfully used to treat a child with an extensive medical history of psychosis who was resistant to treatment [20]. Further research establishing the interplay between organic etiology and psychiatric disorders is needed.

Another possibility would be both SS and SOX1 encephalitis being in a spectrum or an overlapping syndrome. SOX1, aside from its usual association, has been reported in Guillain-Barré syndrome [21], multiple sclerosis [8], neuromyelitis optica [22], and movement disorder [23]. This illustrates that SOX1 may have a variety of clinical manifestations and its presence among psychiatric patients may be part of an overlapping syndrome or spectrum of disorders.

Autoimmune encephalitis preceded by another disease entity, again rare, has been reported. One example would be N-methyl-D-aspartate receptor antibody encephalitis after herpes simplex virus encephalitis [24]. As SOX1 has been identified as a target for autoimmune encephalitis [9], it can cause encephalitis with psychiatric manifestations. Similar to our patient with abnormal mentation and SS, Gong et al. presented a patient who was positive for anti-SOX1 and GABAB antibodies and presented with an atypical case of limbic encephalitis that involved seizures and mental deficiencies [25]. Furthermore, because the immunological therapy resolved the neurological symptoms, this supports our hypothesis that SOX1 may have played a role in the abnormal mentation of our patient with SS.

However, we are far from establishing an actual relationship between SS and SOX1 positivity. Atypical presentation, in this case, altered mental status out of proportion to SS, requires further investigation. Therefore, antibody testing should be considered when there are abnormal symptoms. More studies will be needed to determine the benefit of immunomodulation therapy for psychiatric patients with positive autoimmune antibodies, especially when the patient has limited improvement with usual psychiatric treatment.

## Conclusions

Previously, SOX1 has been identified in paraneoplastic neurological disorders and autoimmune encephalitis. There has been little established association with psychiatric conditions. Here, we discussed our patient who was diagnosed with SS with features of encephalitis and tested positive for SOX1 antibody. The patient improved with cyproheptadine in addition to IVIG. Increased testing for autoimmune neurological antibodies in atypical presentation is highly suggested. More studies are needed to establish if SOX1 truly plays a pathological role in SS or other psychiatric conditions as well as if SOX1-related encephalitis occurs more often with SS or other psychiatric disorders.
